# DNA Methylation Analysis of Growth Differences between Upright and Inverted Cuttings of *Populus yunnanensis*

**DOI:** 10.3390/ijms25105096

**Published:** 2024-05-07

**Authors:** Haiyang Guo, Tiansu Guo, Hailin Li, Shaojie Ma, Xiaolin Zhang, Chengzhong He, Dan Zong

**Affiliations:** 1 Key Laboratory for Forest Genetics and Tree Improvement and Propagation in Universities of Yunnan Province, Southwest Forestry University, Kunming 650224, China; ghy10101205@163.com (H.G.); 619170900@swfu.edu.cn (T.G.); hailin@swfu.edu.cn (H.L.); mashaojie1013@163.com (S.M.); sunnyxlz27@163.com (X.Z.); hecz@swfu.edu.cn (C.H.); 2 Key Laboratory for Forest Resources Conservation and Utilization in the Southwest Mountains of China, Ministry of Education, Southwest Forestry University, Kunming 650224, China

**Keywords:** DNA methylation, *Populus yunnanensis*, inverted cuttings, *DET2* gene

## Abstract

DNA methylation is an important mechanism for epigenetic modifications that have been shown to be associated with responses to plant development. Previous studies found that inverted *Populus yunnanensis* cuttings were still viable and could develop into complete plants. However, the growth status of inverted cuttings was weaker than that of upright cuttings, and the sprouting time of inverted cuttings was later than that of upright cuttings. There is currently no research on DNA methylation patterns in inverted cuttings of *Populus yunnanensis*. In this study, we detected genome-wide methylation patterns of stem tips of *Populus yunnanensis* at the early growth stage and the rapid growth stage by Oxford Nanopore Technologies (ONT) methylation sequencing. We found that the methylation levels of CpG, CHG, CHH, and 6mA were 41.34%, 33.79%, 17.27%, and 12.90%, respectively, in the genome of inverted poplar cuttings, while the methylation levels of the four methylation types were higher in the genome of upright poplar cuttings than in inverted cuttings, 41.90%, 34.57%, 18.09%, and 14.11%, suggesting important roles for DNA methylation in poplar cells. In all comparison groups, CpG-type methylation genes in the Kyoto Encyclopedia of Genes and Genomes (KEGG) pathway were annotated to pathways associated with carbon metabolism, ribosome biogenesis in eukaryotes, glycolysis/gluconeogenesis, pyruvate metabolism, and mRNA detection pathways, suggesting that different biological processes are activated in upright and inverted cuttings. The results show that methylation genes are commonly present in the poplar genome, but only a few of them are involved in the regulation of expression in the growth and development of inverted cuttings. From this, we screened the *DET2* gene for significant differences in methylation levels in upright or inverted cuttings. The *DET2* gene is a key gene in the Brassinolide (BRs) synthesis pathway, and BRs have an important influence on the growth and development process of plants. These results provide important clues for studying DNA methylation patterns in *P. yunnanensis*.

## 1. Introduction

Epigenetics, a phenomenon in which DNA sequences in the genome remain unaltered but gene function is altered and can be inherited and retained through cellular mitosis, was first proposed by Waddington [1]. DNA methylation is a major form of epigenetic modification that exhibits specificity across various plant species, tissues, organs, and developmental stages [2,3,4,5,6]. The process of methylation, which involves the addition of a methyl group to cytosine, takes place at the 5 position on the pyrimidine ring [7]. This is the same position where thymine, a DNA base, has its methyl group. This methyl group is absent in uracil, a similar base found in RNA. The conversion of 5-methylcytosine to thymine occurs through spontaneous deamination [8]. DNA methylation, as one of the most important regulatory phenomena in epigenetics, has crucial functions such as regulating gene expression, maintaining genetic material stability, and establishing epigenetic patterns. It also plays a key role in cellular processes such as transcriptional regulation, transposon inactivation, and genomic imprinting [9]. DNA methylation has emerged as one of the key methods for examining plant growth, development, and resilience to adversity in recent years. Many studies have demonstrated that DNA methylation plays a crucial role in responses to adverse stress conditions [10]. *Chorispora bungeana* responded to cold stresses rapidly through the alterations of DNA methylation to adapt to the intricate cold stresses in the alpine areas [11]. The RNA-directed DNA methylation (RdDM) pathway can dynamically regulate a large number of heat stress response genes [12]; Salt-induced expression of the *AtMYB74* transcription factor is silenced by RdDM under normal conditions but activated after salt treatment [13]. Effective reduction of malonaldehyde (MDA) and reactive oxygen species (ROS) levels, as well as enhancement of antioxidant activity, endogenous melatonin levels, proline, and pigment content, were observed after active modulation of methylation status in specific regions of the genome. In addition, redox and melatonin biosynthesis-related gene expression was upregulated [14]. DNA methylation can play a role in the regulation of oxidative stress and ROS in plants under a variety of abiotic stress conditions, including salt, cold, and heat, by regulating the expression of genes related to redox and melatonin biosynthesis [15]. DME-LIKE 2 (DML2) mediates DNA demethylation during fruit development, and in loss-of-function DML2 tomato mutants, fruits fail to develop to maturity, indicating that DNA methylation patterns affect plant growth and development [16].

*Populus yunnanensis* Dode is a natural tree species endemic to southwest China with excellent characteristics such as fast growth, strong rooting ability, and high survival rate of cuttings, thus becoming one of the most productive timber species in China [17]. Previous studies have shown that *P. yunnanensis* cuttings that are inverted can survive, but their rooting and sprouting time is slower compared to upright cuttings. In addition, the growth of the main branch thickness and seedling height of inverted cuttings is considerably lower than that of upright cuttings [18]. Studies have shown that DNA methylation plays a crucial role in plant responses to adversity stress, and the inverted insertion of poplar cuttings has a certain effect on the growth and survival of cuttings and leads to the re-establishment of auxin transport direction in bark and side buds of inverted cuttings (from lower morphological end to upper morphological end) [8,18,19]. Zhou et al. found that some plant hormones such as growth hormone (IAA), cytokinin (CTK), gibberellins (GAs), ethylene (ET), and brassinosteroids (BRs), changed considerably in inverted cuttings [18]. Given that inverted cuttings may affect the DNA methylation levels of plants, there are currently no reports on DNA methylation in *P. yunnanensis* inverted cuttings. Therefore, this study constructed DNA methylation maps for two *P. yunnanensis* cuttings (upright and inverted cuttings) from Oxford Nanopore Technologies (ONT) and compared the distribution of CG, CHG, CHH, and 6mA methylation levels in different growth stages. This study aims to explore *P. yunnanensis* methylation genome pattern and changes related to inverted stress, as well as identify key genes involved in growth based on methylation patterns.

## 2. Results

### 2.1. Methylation Profiles in P. yunnanensis Stem Tip

The ONT data were generated from inverted and upright samples collected at the initial and rapid growth stages. Following the removal of low-quality data, we obtained 13,772,462 clean reads (MeanQual > 7) from the initial growth stage samples and 14,930,842 clean reads from the rapid growth stage samples for further analysis. Sequence alignment between clean reads of each sample and the *P. yunnanensis* genome was performed, and the alignment efficiency ranged from 98.23% to 99.43% (Table 1). Based on the comparison results and the detection of methylation locus of the original electrical signal, each sample can detect an average of 4,390,229 CpG, 6,796,573 CHG, 32,540,903 CHH, and 38,595,366 6mA locus (10× depth) (Table 2; Figure S1). These loci were distributed across all 19 chromosomes, and the number of loci in each chromosome was correlated with its length, indicating the prevalence of methylation in the poplar genome. Comparing the number of loci in the inverted and upright samples, we found a slight increase in methylation levels in the upright samples. Comparing the number of loci in the samples for each growth stage, we found a slight increase in methylation level in the initial growth stage samples.

**Figure 1:**
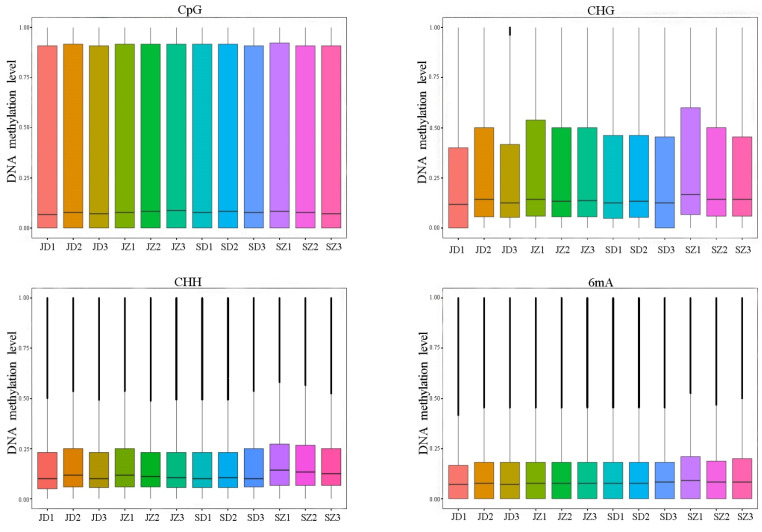
Comparative box plot of the overall distribution of methylation levels for each sample.

**Figure 2:**
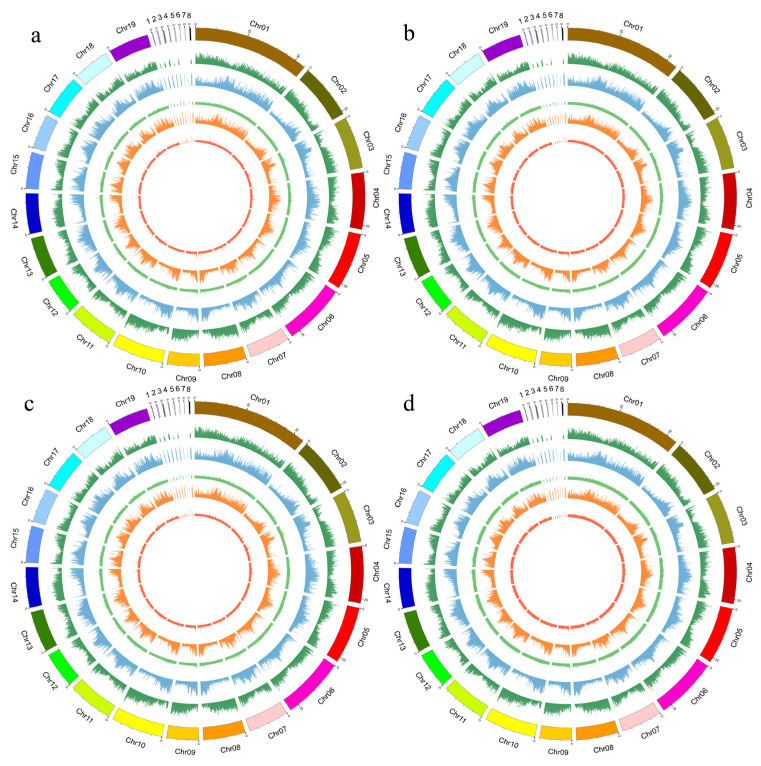
Genome-wide methylation levels of different samples. (**a**) JD (inverted cuttings at the early stage of growth); (**b**) JZ (upright cuttings at the early stage of growth); (**c**) SD (inverted cuttings at the rapid growth period); (**d**) SZ (upright cuttings at the rapid growth period). 1–8: Contig00021, Contig00387, Contig00139, Contig00070, Contig00237, Contig00121, Contig00246, Contig00109; The methylation levels of chromosomes, gene density in windows, CpG, CHH, CHG, and 6mA are indicated from outside to inside.

The methylation levels of the CpG site in the genome of Py-JZ (upright cuttings at the early stage of growth) were 42.30%, the CHG site was 33.99%, the CHH site was 17.25%, and the 6mA site was 11.73%, indicating the percentage of methylation levels in the *P. yunnanensis* genome. Correspondingly, Py-JD (inverted cuttings at the early stage of growth) had methylation levels of 40.95%, 33.43%, 17.11%, and 12.69% in the CG, CHG, CHH, and 6mA sequence contexts, respectively. We can see the difference in methylation levels between inverted and upright cuttings. The genomic methylation level of Py-SZ (upright cuttings at the rapid growth period) was 41.52% CG, 35.15% CHG, 18.92% CHH, and 15.50% 6mA. Correspondingly, Py-SD (inverted cuttings at the rapid growth period) had methylation levels of 41.73%, 34.15%, 17.43%, and 13.10% in the CG, CHG, CHH, and 6mA sequence contexts, respectively. In examining the distribution of methylation sites in the four sequence contexts, we found that the highest methylation levels were found at the CpG site, followed by the CHG site, and the lowest methylation levels were found at the CHH and 6mA sites. The four methylation types in the genomes of *P. yunnanensis* upright cuttings were slightly higher than those of inverted cuttings, and CpG methylation was the major methylation type in both upright and inverted cuttings of *P. yunnanensis* (Figure 1). Genome-wide methylation analysis showed that the methylation levels of the CpG locus and CHG locus were significantly higher than those of the CHH locus and 6mA locus and were negatively correlated with gene density (Figure 2).

**Figure 3:**
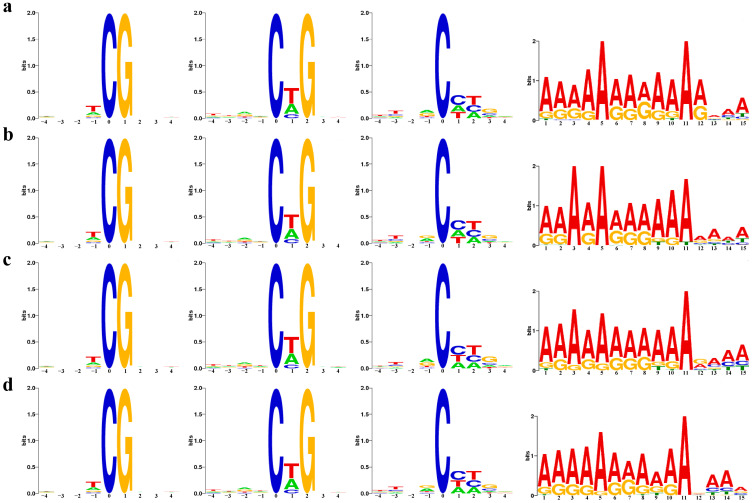
Sequence distribution around the 5 mC site of samples and motif distribution in the 6 mA region of samples. From left to right, CG, CHG, CHH and 6 mA. (**a**) JD; (**b**) JZ; (**c**) SD; (**d**) SZ.

In all mCGs and mCHH, the DNA sequence logo of methylated cytosine showed a consistent bias towards the sequence, regardless of whether it was inverted cuttings or normal cutting. Similarly, the DNA sequence logo of methylated adenine showed consistent bias towards the sequences in all 6mA sites (Figure 3).

After two-way ANOVA analysis of methylation differences, the results showed that in CpG methylation, there was a significant difference in the effect of upright and inverted cuttings on methylation levels (F = 8.656, *p* < 0.05), with upright cuttings having significantly higher methylation levels than inverted cuttings (*p* < 0.05). However, in CHG, there was no significant difference (*p* > 0.05) between upright and inverted cuttings of different growth periods on methylation levels. In CHH, the methylation level of upright cuttings was significantly higher than that of inverted cuttings (F = 6.797, *p* < 0.05), and the methylation level of the fast-growing stage was highly significantly higher than that of the primordial stage (F = 16.926, *p* < 0.01), and there was an interaction between the upright and inverted cuttings and the different growth periods (F = 7.621, *p* < 0.05). In 6mA, the methylation level of upright cuttings was significantly higher than that of inverted cuttings (F = 9.222, *p* < 0.05), and there was no significant difference between different growth periods. In summary, we hypothesize that there are methylation differences between upright and inverted cuttings of *P. yunnanensis*.

The RepeatMasker [20] was used to predict the methylation levels in the repeat regions, and the results showed that the four samples showed hyper-methylated in the repeating region and hypo-methylated in the upstream and downstream regions of CpG and 6mA methylation types. A similar pattern was observed in the CHG and CHH methylation types, where the repeating regions showed hyper-methylated and the upstream and downstream regions showed hypo-methylated, but the downstream regions had slightly higher methylation levels than the upstream regions (Figure S2).

**Figure 4:**
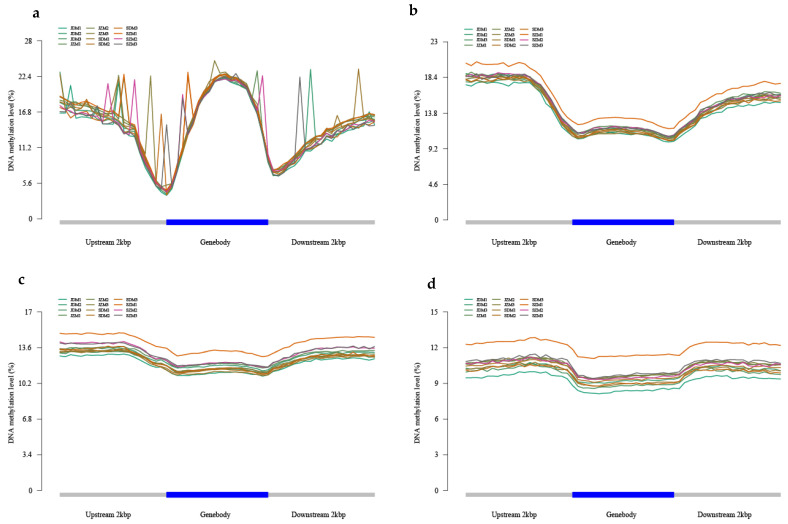
Methylation levels in different regions of all genes. (**a**) CG; (**b**) CHG; (**c**) CHH; (**d**) 6mA.

Based on the location of the methylation locus on the reference genome and the gene location information on the reference genome, we found that all samples showed hypo-methylation levels in the genomic region and hyper-methylation levels in the upstream promoter region and downstream region. Within the genic regions, the methylation loci were most often located in the promoters (Figure 4). The methylation locus distribution patterns of *P. yunnanensis* in genic regions were similar to those of *Populus nigra* and *Populus. trichocarpa* [21].

### 2.2. Methylation Changed in Different Growth Stages and Cutting Methods in P. yunnanensis

**Figure 5:**
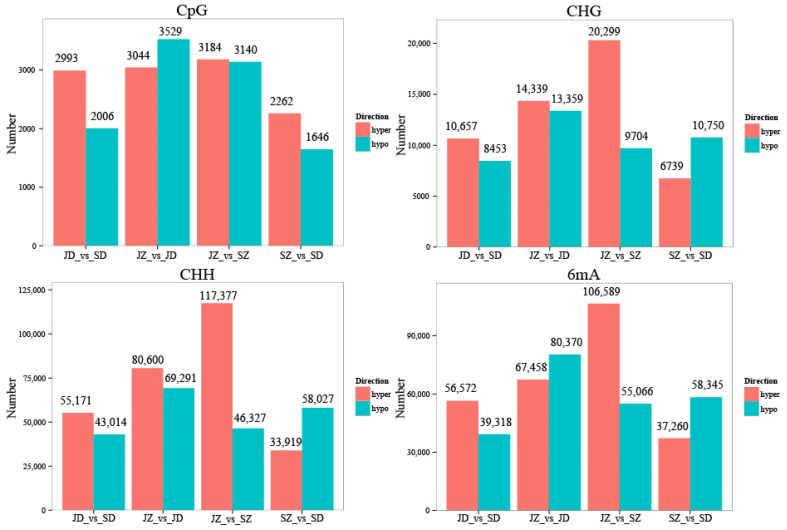
Statistics of DML of samples.

In the present study, a total of 218,184, 331,990, 361,686, and 208,948 DMLs were identified respectively in the four comparison groups: JD_vs_SD, JZ_vs_JD, JZ_vs_SZ, and SZ_vs_SD (Figure 5). When differential methylation loci (DMLs) were annotated to the reference genome, it was observed that the CpG methylation locus was mainly located in the Distal Intergenic (approximately 49.70~56.57%). Within the genic regions, the methylation loci were most often located in the promoters (approximately 33.81~38.61%). The CHG methylation loci were mainly located in the distal intergenic (approximately 20%) and promoters (>50%). The distribution of CHH methylation sites is the same as that of CHG methylation. The 6mA methylation loci were mainly located in the distal intergenic (31~36%) and promoters (45~49%) (Figure S3). If a DML annotates a non-intergenic region of a gene, the gene is considered to be an associated gene of the DML. Gene ontology (GO) analysis of these associated genes was performed by AgriGO to detect the biological nature of the DNA methylation in the poplar genome. The most enriched GO terms in the upright and inverted samples were similar, such as cellular process, metabolic process, single–organism process, and biological regulation in the biological process GO domain; cell, cell part, organelle, membrane, membrane part, organelle part in the cellular component GO domain; binding, catalytic activity, transporter activity, nucleic acid binding transcription factor activity, structural molecule activity in the molecular function GO domain (Figure S4).

**Figure 6:**
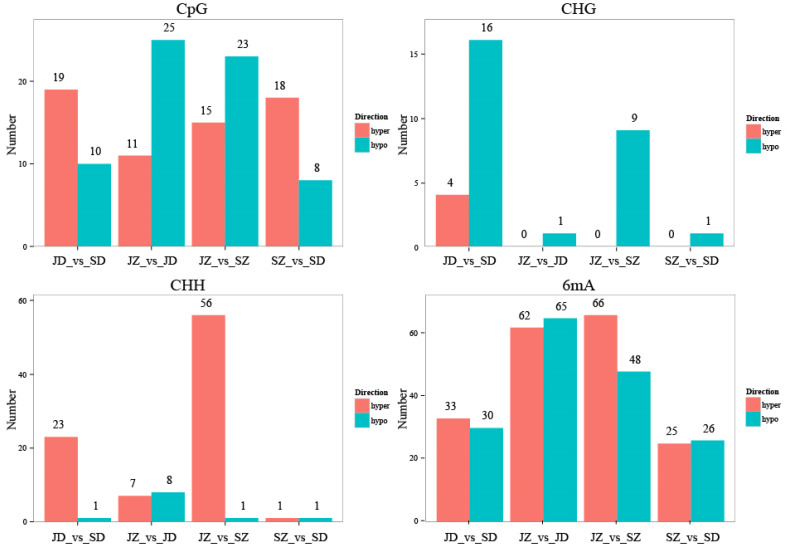
Statistics of the DMR of the samples.

**Figure 7:**
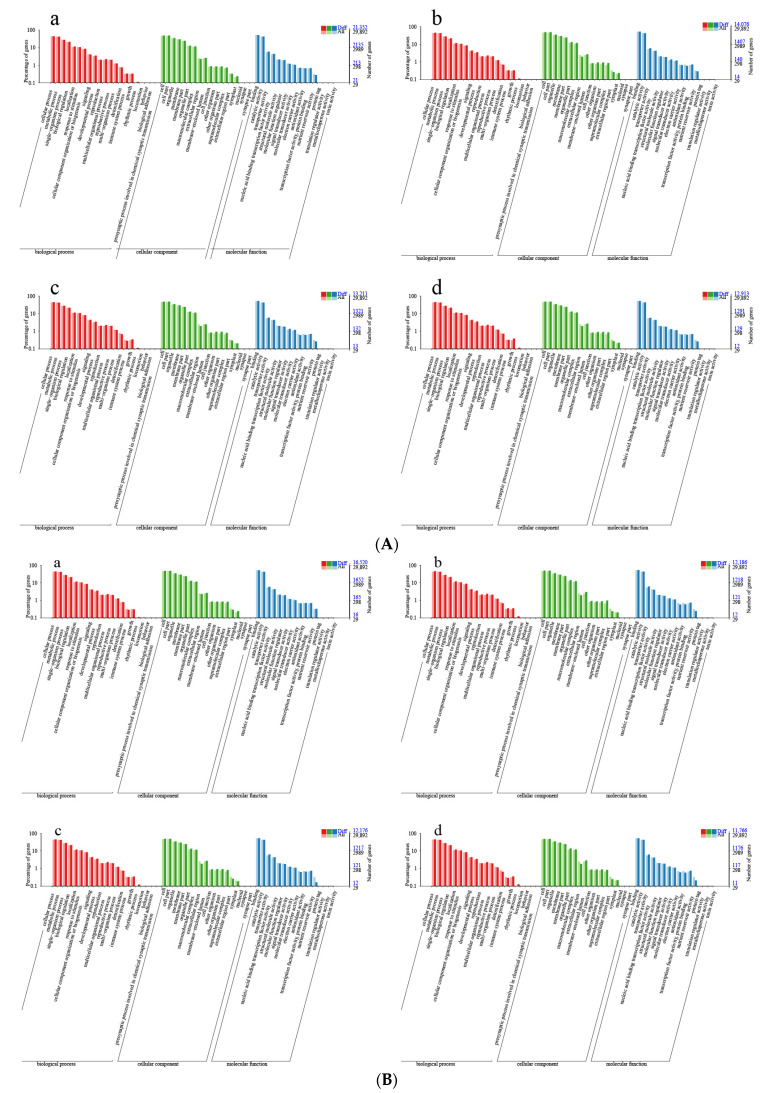
(**A**) GO enrichment map of DMR-associated genes in comparison group JZ_vs_JD. (**a**) CG; (**b**) CHG; (**c**) CHH; (**d**) 6mA; (**B**) GO enrichment map of DMR-associated genes in comparison group SZ_vs_SD. (**a**) CG; (**b**) CHG; (**c**) CHH; (**d**) 6mA.

**Figure 8:**
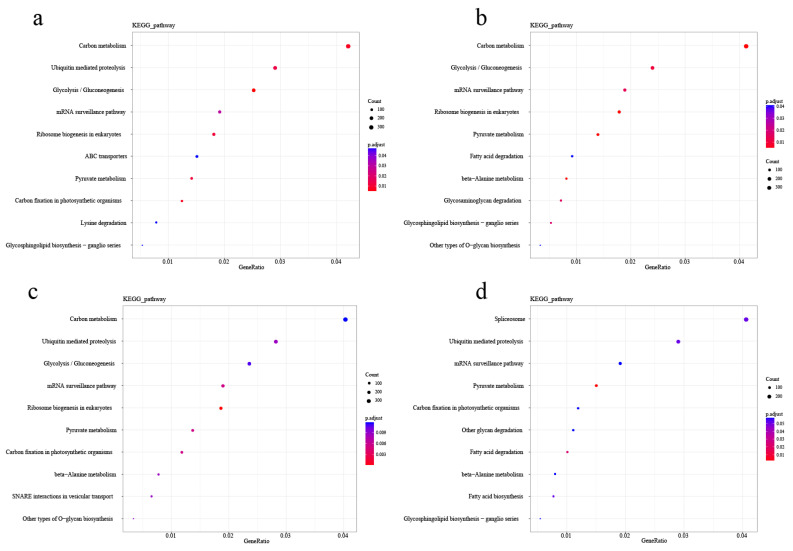
GO enrichment map of DMR-associated genes of CpG type. (**a**) JD_vs_SD; (**b**) JZ_vs_JD; (**c**) JZ_vs_SZ; (**d**) SZ_vs_SD.

A total of 136, 178, 218, and 80 differential methylation regions (DMRs) were identified in the four comparison groups, respectively (Figure 6). When DMRs were annotated to the reference genome, it was observed that the CpG methylation loci were mainly located in the distal intergenic (approximately 30%). Within the genic regions, the methylation loci were most often located in the promoters (>50%). The CHG methylation loci were mainly located in the distal intergenic (29~33%) and promoters (>47%). The CHG methylation loci were mainly located in the Distal Intergenic (35%) and promoters (4~51%). The 6mA methylation loci were mainly located in the Distal Intergenic (approximately 41%) and promoters (40~45%) (Figure S5). To better describe the corresponding biological function of these DMRs, we identified DMRs that overlapped with gene regions; the distribution of DMRs in genic regions was similar to that of the locus. Gene ontology (GO) analysis of these associated genes was performed by AgriGO to detect the biological nature of the DNA methylation in the poplar genome. The enrichment results were the same as those of DML. The most enriched GO terms were cellular process, metabolic process, single–organism process, and biological regulation in the biological process GO domain; cell, cell part, organelle, membrane, membrane part, organelle part in the cellular component GO domain; binding, catalytic activity, transporter activity, nucleic acid binding transcription factor activity, structural molecule activity in the molecular function GO domain (Figure 7). The KEGG pathway analysis indicated that all the CpG methylated genes were mapped to carbon metabolism, ribosome biogenesis in eukaryotes, glycolysis/gluconeogenesis, pyruvate metabolism, and mRNA detection pathways. In addition, CpG methylated genes in JZ_vs_JD, JD_vs_SD, and JZ_vs_SZ comparison groups were annotated to ubiquitin-mediated proteolysis in KEGG (Figure 8). Therefore, we speculated that a few genes changed DNA methylation status, affecting the growth of poplar.

The accuracy of ONT was validated by bisulfite sequencing PCR (BSP) using the DNA samples for ONT. Six candidate genes were screened by combined analysis of methylation sequencing data as well as transcriptome sequencing data (GSE249347), including *Pyun02G001640* (mitogen-activated protein kinase organizer 1), *Pyun09G000880* (superoxide dismutase, Cu-Zn family), *Pyun03G003680* (uncharacterized protein), *Pyun08G000970* (steroid 5-alpha-reductase), *Pyun17G009050* (transcription factor MYB, plant) and *Pyun18G012180* (S-(hydroxymethyl) glutathione dehydrogenase/alcohol dehydrogenase).

**Figure 9:**
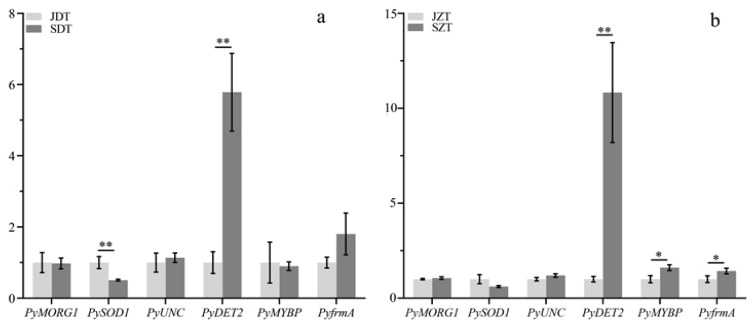
Relative expression of genes. (**a**) inverted; (**b**) upright. “*” indicates significant differences (*p* < 0.05); “**” indicates highly significant differences (*p* < 0.01).

The expression levels of the six genes were detected using qPCR. The expression levels of the *Pyun08G000970* (steroid 5-alpha-reductase) were significantly upregulated in the rapid growth stage compared to the early growth stage. And the relative expression of the *Pyun08G000970* gene at the rapid growth stage was 10.8 times that at the early growth stage in the upright samples; the relative expression of *Pyun08G000970* gene at the rapid growth stage was 5.7 times that at the early growth stage in the inverted samples (Figure 9).

### 2.3. Sequence Analysis of PyDET2 Gene

**Figure 10:**
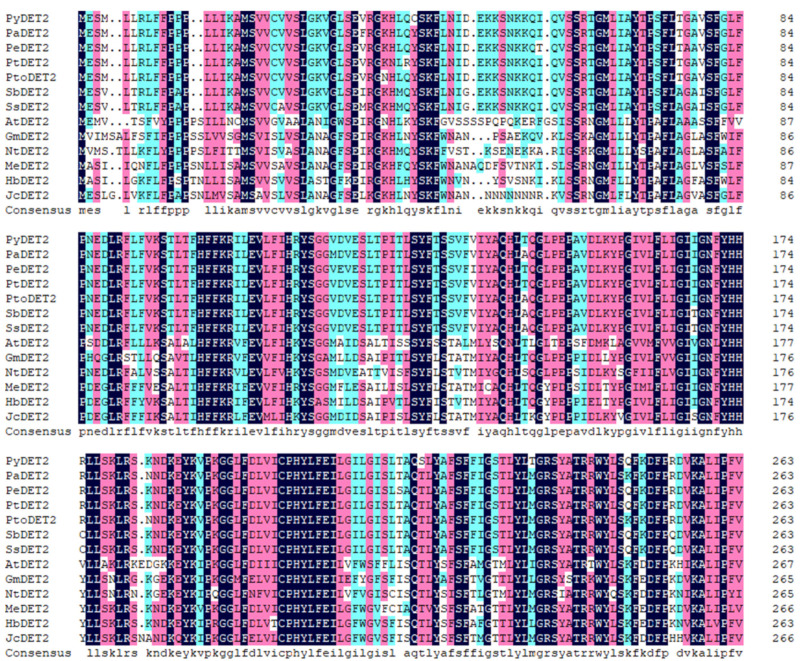
Comparative analysis of homologous amino acid sequences of *DET2* in different plants.

We cloned and analyzed the sequence of the *PyDET2* gene and performed preliminary functional studies. The amino acid sequence of *PyDET2* was analyzed using Expasy-ProtParam tool (http://web.expasy.org/protParam/, accessed on 20 December 2022). The results showed that the amino acid sequence of the gene had a molecular weight of 30,020.48 Da, a theoretical isoelectric point (pI) of 9.66, an aliphatic index of 108.11, and a grand average of hydropathicity (GRAVY) of 0.340. Analysis of the NCBI-CDD structural domain indicated the presence of a PEMT superfamily structural domain in this gene. Sequence alignment analysis using DNAMAN software (v6.0) showed that the amino acid sequence of *DET2* in *P. yunnanensis* showed similarity to that of *Populus alba*, *Populus euphratica*, *Populus trichocarpa*, *Populus tomentosa*, *Salix brachista*, *Salix suchowensis*, *Arabidopsis thaliana*, *Glycine max*, *Nicotiana tabacum*, *Manihot esculenta*, *Hevea brasiliensis* and *Jatropha curcas*, and the similarity with poplar species was as high as 97.62%. The high similarity of *DET2* amino acid sequences with poplar species was 97.62%, indicating that the gene has a clearly conserved region (Figure 10).

**Figure 11:**
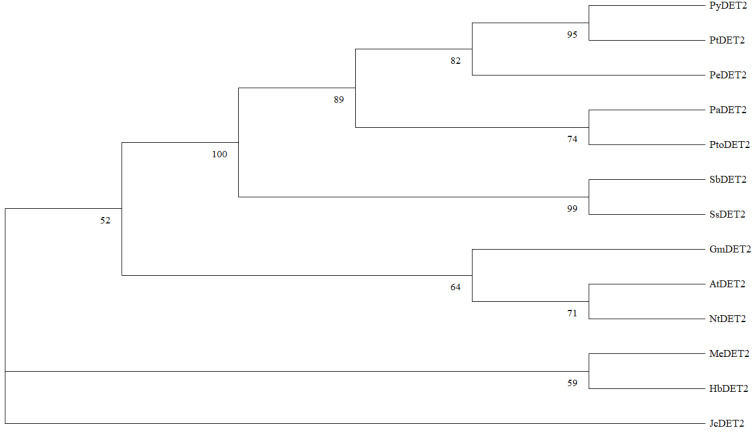
Phylogenetic tree of *DET2* homologous amino acid sequences of different plants.

A phylogenetic tree (Bootstrap = 1000) was constructed for the *DET2* amino acid sequences of *P. yunnanensis*, *P. alba*, *P. euphratica*, and *P. trichocarpa* using the neighbor-joining method with MEGA 7 software. The results showed that the gene is highly related to Populus and that *P. yunnanensis* and *Populus trichocarpa* are in the same phylogenetic branch, and Populus species are also closely related to *M. esculenta*, *H. brasiliensis*, and *J. curcas*, which are species of Euphorbiaceae (Figure 11).

*PyDET2* gene promoter sequences were imported into the PlantCARE and PlantTFDB online websites for cis-acting element prediction analysis. On the *PyDET2* gene promoter’s positive and negative strand sequences, we discovered several cis-acting elements. There are additional cis-acting elements associated with light response and stress tolerance in addition to the transcriptionally necessary CAAT-box and TATA-box. A cis-element involved in the abscisic acid response (ABRE) is located at +1875 bp. A cis-element involved in defense and stress response (TC-rich repeats) is located at +1360 bp, a cis-element involved in light response (G-Box) is located at −1874 bp, a cis-element related to hyphal tissue expression (CAT-Box) is located at −412 bp, a cis-element involved in gibberellin response (P-Box) is located at +1038 bp, a region involved in light response module is located at −1970 bp, two cis-element involved in salicylate response cis-elements at −1126 bp and −1753 bp, two regions of light-responsive conserved DNA modules at +121 and −1215 bp, four cis-elements involved in the low-temperature response at −752, −1172, −1235, and −1522 bp, and seven regulatory cis-elements involved in anaerobic induction at −601, −669, +1202, +1207, +1298, +1675, and +1927 bp (Table 3).

## 3. Discussion

The characterization of genome-wide methylation patterns in plant systems has been a hot research area in recent years. In previous studies, it has been found that inversion cutting has certain effects on the synthesis of endogenous hormones and signal transduction of *P. yunnanensis* [18,22]. The present study is the first to systematically compare genome-wide methylation profiles in *P. yunnanensis* shoot apex between upright and inverted. Using the ONT method, we obtained DNA methylation profiles for both kinds of cuttings, and numerous genes were found to be methylated in different genic regions, especially in the promoter regions, it is suggested that DNA methylation may have a potential role in regulating gene downstream. These genes were shown to be involved in multiple biological processes, molecular functions, and cellular components, indicating the importance of the roles of DNA methylation in *P. yunnanensis*.

Additionally, the methylation levels of CpG, CHG, CHH, and 6mA in the genome of the inverted seedling of *P. yunnanensis* were 41.34%, 33.79%, 17.27%, and 12.90%, respectively. However, the methylation levels of CpG, CHG, CHH, and 6mA in the genome of the upright seedling of *P. yunnanensis* were 41.90%, 34.57%, 18.09%, and 14.11%. Therefore, we know that a small part of genes changed their methylation state due to different cutting methods and CpG methylation was the main methylation type in *P. yunnanensis*. In *P. yunnanensis* upright and inverted cuttings, DNA repeat regions, CpG island regions, and non-coding regions showed hypermethylation levels, while hypomethylation levels were found in gene body regions, which was more consistent with the result that gene body methylation was positively correlated with gene expression in rice [23,24]. CpG and CHG methylation levels were positively correlated with DNA repeat regions and negatively correlated with gene density, and the results suggest that DNA methylation has a function in maintaining genomic stability [25,26].

Brassinolide (BRs), a crucial plant hormone, are found in all plant organs and play a key role in a variety of growth and development processes, including seedling growth, cell elongation, cell wall formation, and the way that plants react to environmental stresses like drought, low temperature, and high salinity. Fujioka et al. elucidated the basic pathway of BR synthesis by a suspension-cultured periwinkle cell system using campesterol (CR) as the initiator of BR synthesis [27]. The *DET2* gene encodes a steroid 5α-reductase, which is a key rate-limiting enzyme in BR biosynthesis, and deletion of the *DET2* gene in *Arabidopsis thaliana* leads to leaf de-yellowing, enhanced antioxidant activity, and affects root elongation growth [28,29].

Identification and analysis of the *PyDET2* family showed that the *PyDET2e* (*PyDET2*) gene has a CDS length of 795 bp, encodes 264 amino acids, and has a protein molecular weight of 30.02 kDa. It is an unstable hydrophobic protein without a signal peptide, with three transmembrane regions consisting of α-helices, and is presumably a non-secretory membrane protein, with a hydrophobicity index of 0.34, which is consistent with the characteristics of membrane-localized proteins. The vector was constructed and transferred into tobacco leaves for observation, and it was found that the protein was distributed in chloroplasts, so it was presumed to be a chloroplast membrane-bound protein [30]. The presence of the Steroid_dh(pfam02544) structural domain (which belongs to the PEMT superfamily) was shown by NCBI-CDD structural domain analysis. In studies with *A. thaliana*, it was found that functional deficiency of the det2 gene resulted in a significant reduction in the cellulose synthase gene and the content of cellulose [31].

The *PyDET2* gene was successfully cloned in *P. yunnanensis*, and sequence comparison analysis revealed that the protein sequence encoded by the *PyDET2* gene is highly similar to that of Salicaceae (97.62%) and has high similarity with the *DET2* protein sequence in Euphorbiaceae. This suggests that the protein encoded by the *PyDET2* gene is relatively conserved in evolutionary terms. Wang et al. introduced a *DET2* gene overexpression vector into poplar (*P. alba × P. glandulosa*), and *PagDET2* overexpression lines promoted apical and radial growth, thus enhancing stem height and stem diameter in the transgenic trees [32]; Li et al. successfully cloned the 84 K poplar *PagDET2* gene and transformed 84 K poplar using the Agrobacterium-mediated method, and the transgenic poplar. The endogenous BR content of transgenic poplar was significantly higher than that of wild type 84 K poplar, and overexpression of the *DET2* gene promoted high growth of 84 K poplar [33]. In one study, the *DET2* overexpression gene vector was introduced into *P. tomentosa*, which promoted the elongation and thickening of *P. tomentosa* stems and the growth of adventitious roots [34]. *GmDET2* was able to significantly promote the increase of hypocotyl length and plant height in *A. thaliana* [35]. Based on this, we hypothesized that *PyDET2* may influence the tall and thick growth of *P. yunnanensis*.

The analysis of cis-acting elements in the promoter region of the *PyDET2* gene of *P. yunnanensis* showed that there were more light-responsive and anti-adversity-related elements in the promoter region, and it was hypothesized that the expression of the PyDET2 gene might be regulated by light or stress. It has been discovered that light signaling and brassinolide work in concert. The transcription factor GATA2 interacted with the BR and light signaling pathway to restore the light form after exposing yellowed plants to light settings [36]. The synergistic action of BR and light signaling promotes growth processes in *Arabidopsis*, including the expression of light-regulated photosynthetic genes, cell elongation, normal leaf and chloroplast senescence, and flower induction [37,38]. It indicates that *DET2* gene expression with light signaling works in concert, but more research is required to determine the precise process.

Previous studies have shown that the synthesis and signal transduction of endogenous hormones in *P. yunnanensis* were affected after inversion; the synthesis of the coding for USPase was affected, thus affecting the anabolism of glycans in the cell wall, and these may be one of the factors that hinder the growth and development of inverted cuttings [18,22]. In our study, five growth-related genes were identified by ONT sequencing, among which *DET2* gene differential levels were the most significant. And we found that the expression of *the DET2* gene in upright cuttings was higher than that in inverted cuttings, so we speculated that it might be due to the synthesis of BRs caused by the differential expression of *the DET2* gene, which led to the differential growth of orthotropic and inverted cuttings of *P. yunnanensis*. In conclusion, the *DET2* gene may play an important role in the tall growth and stem elongation of plants, but further studies are needed to determine whether the *PyDET2* gene has the same effect in *P. yunnanensis*.

## 4. Materials and Methods

### 4.1. Plant Material Cultivation and Collection

Three one-year-old *P. yunnanensis* clones from the Haikou Forest Farm of Kunming (Kunming, China) were selected as three biological replicates, and their main stems were used to generate cuttings of similar length and diameter. We cultivated the cuttings at Southwest Forestry University (Kunming, China. 102.76 E, 25.06 N) during June in two orientations: upright and inverted. Stem tip parts of the main branches were collected in July (early growth period) and September (rapid growth period), and three biological replicates were taken for each condition. The sample groups from two periods June (J) and September (S) of the upright (Z) and inverted (D) cuttings were named JZ, JD, SZ, and SD, and the three replicates within each group were numbered with 1 (e.g., JZ1), 2 (e.g., JZ2), and 3 (e.g., JZ3). The samples were rapidly frozen in liquid nitrogen, placed in an ultra-low temperature freezer at −80 °C, and sent to Biomarker Technologies for methylation sequencing analysis.

### 4.2. Data Filtering and Methylation Level Analysis

The raw downstream data format of Nanopore sequencing is binary fast5 format (containing all raw sequencing signals), and a single read corresponds to a single fast5 file. Each read corresponds to a single fast5 file. After basecalling by Guppy software (v6.4.6), the fast5 format data are converted to FASTQ format for subsequent quality control analysis. The raw FASTQ data were further filtered for splices, short fragments (Length < 50 bp), and low-quality reads (MeanQual < 7) to obtain the total dataset. Clean reads were compared to the reference genome by the split-reads (SR) method using minimap2 software [39]. Nanopolish (v0.8.4) is used to detect CpG based on the hidden Markov model [40]. The algorithm adds M bases as methylated C sites, defines the base strings to be detected for methylation status in the reference genome as SRs (at least one CpG site and its upstream and downstream 5 bp base strings are included), and then compares the basecalling reads with the reference genome, and determine whether the SRs are covered by reads or not, and the likelihood value was used to determine whether it was a methylation state. The sequencing data were first re-squiggled using Tombo software (v1.5.1), and then the Alternative Model was used to detect CHH, CHG, and 6mA sites [41]. Methylation levels are more realistic at higher sequencing depths, so C and A sites above the 10× depth of detection are retained for subsequent analysis. The repeat region was predicted using RepeatMasker software (v4.1.5), and the repeat region, upstream 2 Kb, and downstream 2 Kb were divided into 50 bins, and the average methylation level of each bin was counted [20].

### 4.3. Screening of Differential Methylation Regions

SMART2 software was used to screen the differential methylation locus and regions. Differential methylation regions (DMR) were detected based on genomic segmentation, which is a subset with a high methylation similarity locus. Adjacent fragments with similar patterns were further combined into larger fragments. Differential methylation regions (DMR) were analyzed by SMART2 software (v2.2.8) to calculate the *p*-value and difference levels between comparison groups. When *p* < 0.05 for the difference between the read numbers of each methylated region for two samples, this study determined that the result was significant. Methyl Specificity was combined with *p*-value to determine whether a region had differential methylation. The ChIPseeker software (v1.40.0) package was used to annotate the DMR to different gene regions according to the location of the DMR. The methylated exon, intron, promoter, coding sequence (CDS), the 5′-untranslated region (UTR), 3′ UTR, and intergenic region were identified based on P. yunnanensis genome database transcript annotations. If DMR is annotated to a non-intergenic region of a gene, the gene is considered to be an associated gene of DMR. GO/KEGG enrichment analysis of DMR-associated genes was performed by R clusterProfiler (v4.1.1).

### 4.4. Bisulfite Sequencing of Differentially Methylated Genes

To validate the ONT results, DMRs overlapping in six genes were confirmed using bisulfite PCR (BS-PCR) and RNA-Seq. Bisulfite conversions were completed using the DNA Bisulfite Conversion Kit (TIANGEN Biotech Co., Ltd., Beijing, China). Bisulfite-modified PCR primers were designed using the online program MethPrimer (http://urogene.org/methprimer2/, accessed on 19 June 2022). Information on the amplified methylated regions and primers is listed in Table 4. The disulfite-transformed DNA was used as the template DNA for PCR amplification with the TaKaRa EpiTaq HS enzyme (R110Q). The PCR amplification conditions were: 5 min at 94 °C, followed by 40 cycles of 30 s at 94 °C, 30 s at 54 °C and 30 s at 72 °C, and further elongation at 72 °C for 10 min using an ABI Veriti 96-Well Thermal Cycler (Applied Biosystems, Waltham, MA, USA). The PCR products were separated and purified with a DNA product purification kit (TaKaRa, Tokyo, Japan) and a TaKaRa MiniBEST Agarose Gel DNA Extraction Kit Ver.4.0. Purified amplicons were cloned into the pMD 18-T Easy vector (6011) and Trans1-T1 receptor cells (CD501) were used to transform the vector and culture monoclonal colonies. The monoclonal colonies were selected and cultured for positive cloning identification and sequencing. The sequencing results and original sequences were analyzed via the online website QUMA (http://quma.cdb.riken.jp/, accessed on 10 July 2022).

### 4.5. RNA Isolation and qPCR Analyses

Total RNA was extracted from the shoot apex using a Plant RNA Kit (Omega Bio-Tek, Norcross, GA, USA) for gene expression validation experiments. Fast King RT Kit (TIANGEN Biotech Co., Ltd., Beijing, China) was used to synthesize the first cDNA strand of RNA. PD-E1 was used as the internal reference gene for RT-qPCR verification, and primers of the screened genes were designed by Primer5.0 software and NCBI Primer-BLAST. Amplification lengths and primers are listed in Table 5. We performed qPCR analysis in the Rotor-Gene Q (QIAGEN, Duesseldorf, Germany). Each 20 μL PCR system contained 2 μL of first-strand cDNA, 200 nM of primers, and a 2× chamQ SYBR qPCR master mixture (Vazyme, Nanjing, China). The amplification conditions were: 3 min at 95 °C, followed by 40 cycles of 10 s at 95 °C, 30 s at 56 °C and 30 s at 72 °C, and further elongation at 72 °C for 10 min. We performed three replicates for each sample. Relative quantification values were calculated using the 2^−ΔΔCT^ method.

### 4.6. Functional Analysis of PyDET2 Gene

Physicochemical properties of the *PyDET2* gene-encoded protein were analyzed using Expasy-ProtParam (https://web.expasy.org/protparam/, accessed on 20 December 2022) online software. The NCBI online website was used to query the *DET2* amino acid sequences of *Populus alba*, *Populus euphratica*, *Populus trichocarpa*, *Populus tomentosa*, *Salix brachista*, *Salix suchowensis*, *Arabidopsis thaliana*, *Glycine max*, *Nicotiana tabacum*, *Manihot esculenta*, *Hevea brasiliensis* and *Jatropha curcas*. The homologous amino acid sequences of *DET2* from different plants were analyzed by multiple sequence comparisons and the conserved structure of the protein was analyzed using DNAMAN software (v6.0). The phylogenetic tree of *DET2* homologous amino acid sequences from different plants was constructed using MEGA 7 software with the neighbor-joining method. The sequence of 2000 bp upstream of the start codon ATG in the CDS region of the *PyDET2* gene was imported into the PlantCARE (http://bioinformatics.psb.ugent.be/webtools/plantcare/html/, accessed on 22 December 2022) and PlantTFDB (http://planttfdb.gao-lab.org/index.php, accessed on 22 December 2022) online websites for promoter sequence analysis.

## Figures and Tables

**Table 1:** Statistics of clean reads comparison results of each sample.

ID	SeqNum	MeanQual	Mapped	Unmapped	Ratio (%)
JD1	1,907,103	9.07	1,893,080	14,023	99.26
JD2	2,263,391	9.00	2,248,376	15,015	99.34
JD3	2,561,229	9.07	2,541,902	19,327	99.25
JZ1	2,383,091	9.07	2,363,922	19,169	99.20
JZ2	2,382,629	9.55	2,366,818	15,811	99.34
JZ3	2,275,019	9.51	2,262,031	12,988	99.43
SD1	2,462,841	9.54	2,431,027	31,814	98.71
SD2	2,615,083	9.55	2,588,305	26,778	98.98
SD3	2,090,749	9.00	2,056,227	34,522	98.35
SZ1	3,101,285	9.08	3,054,641	46,644	98.50
SZ2	2,414,738	8.92	2,372,104	42,634	98.23
SZ3	2,246,146	9.06	2,216,803	29,343	98.69

**Table 2:** Statistics of different types of methylation sites of each sample.

ID	CpG	CHG	CHH	6mA
JD1	3,762,802	5,329,392	24,840,600	27,585,968
JD2	4,737,441	7,551,680	36,421,035	43,349,990
JD3	4,203,995	6,893,669	33,014,063	38,648,190
JZ1	5,566,422	8,458,870	41,736,818	51,431,038
JZ2	5,207,927	7,815,594	38,080,584	45,926,680
JZ3	5,301,295	7,661,277	37,110,631	44,386,235
SD1	4,332,887	6,400,612	30,245,830	34,609,545
SD2	4,819,742	7,128,564	34,175,017	40,030,356
SD3	2,566,496	4,121,957	18,423,471	19,866,134
SZ1	5,556,780	9,377,372	46,555,303	60,528,769
SZ2	2,992,772	5,201,162	23,789,123	26,780,834
SZ3	3,634,195	5,618,731	26,098,363	30,000,662

**Table 3:** Enumeration of cis-acting elements on the PyDET2 gene promoter.

Element	Sequence (5′-3′)	Location	Number of Elements	Function
ABRE	ACGTG	1875+	1	Abscisic acid responsiveness cis-element
LTR	CCGAAA	752−, 1172−, 1235−, 1522−	4	Low-temperature responsiveness cis-element
TC-rich repeats	ATTCTCTAAC	1360+	1	Defense and stress responsiveness cis-element
TCA-element	CCATCTTTTT	1126−, 1753−	2	Salicylic acid responsiveness element
ARE	AAACCA	601−, 669−, 1202+, 1207+, 1298+, 1675+, 1927	7	Anaerobic induction regulatory cis-element
G-Box	CACGTT	1874−	1	Light responsiveness cis-element
CAT-box	GCCACT	412−	1	Meristem expression-related cis-element
CAAT-box	CCAAT	667−, 834−, 889−, 1184+, 1187−, 1369+, 1378+, 1432−, 1462−, 1560+, 1724+	11	Promoter and enhancer regions cis-element
TATA-box	TATA	205+, 211−, 212+, 217−, 219+, 625−, 626−, 627−, 628+, 1436−, 1443+, 1444−, 1479−, 1489−, 1490−, 1705−, 1706−, 1707−, 1708−, 1743−, 1775−, 1776−, 1777−, 1778−, 1779+, 1780−	26	Core promoter element around −30 of transcription start
P-box	CCTTTTG	1038+	1	Gibberellin-responsive element
Box 4	ATTAAT	121+, 1215−	2	Part of a conserved DNA module involved in light responsiveness
AE-box	AGAAACTT	1970−	1	Part of a module for light response
Sp1	GGGCGG	180−	1	Light-responsive element
GT1-motif	GGTTAA	1815−	1	Light-responsive element
TCCC-motif	TCTCCCT	92−	1	Part of a light-responsive element
GA-motif	ATAGATAA	1393−	1	Part of a light-responsive element
TCT-motif	TCTTAC	1501+	1	Part of a light-responsive element

**Table 4:** Primer sequence of BSP.

Name	Primer Sequence	Amplification Length	Site (to TSS)
*PyMORG1*	F: GGTTTTGAGTTAAAATAAATAATTAAATTA	191	−534
	R: ACCAAAATTTCTCAAAAAATAAACAC		
*PySOD1*	F: TTAAAAGTTGTTATTATTAGATTGAAGGTT	207	−4055
	R: ACCACAATAATATTATCCCCTCTTC		
*PyUNC*	F: AGGTGTTTTTTAAGAAGAGGTATTTATATT	155	+38,126
	R: T AAAAAATCATCCTTCCACTATACCTC		
*PyDET2*	F: TGTGGGTTATAATTAGGGGTGTTTA	234	−2069
	R: AATTAATATTTAATATCAAATTTTTCCTTC		
*PyMYBP*	F: TTATTTGGGTTTGTATTTATATATGTGAGA	254	+22,128
	R: TCCTTTCAAAACCACTAAAAATTTAC		
*PyfrmA*	F: ATTGGTTTAGTTGTTAGGATAAAGG	111	−2027

**Table 5:** Primer sequence of RT-qPCR.

Name	Primer Sequence	Amplification Length/bp
*PD-E1*	F: ATGAGAACTGGTGGTATTGGTGC	164
	R: GTCACAATCTGGGCAGGTTGAAC	
*PyMORG1*	F: GAGCCCCAGAAGCAGACAAT	188
	R: CCCGGAACATCCTCGGAAAT	
*PyUNC*	F: GATGTCAGGGCTGCCTACG	188
	R: TAAAGGCTCCTGCTCCATGTG	
*PyDET2*	F: TACAAGGTTCCCAAGGGTGG	154
	R: AACTCCTCCCCGTCAGGTAA	
*PySOD1*	F: CCTCATGGATTCCACCTGCAT	174
	R: TATTATTGCCTCTGCCACCCC	
*PyMYBP*	F: GGTCCTGGGAATTGGAGAGC	166
	R: TGTTGCCTAGAAGGGCTTGG	
*PyfrmA*	F: GCAAGGGGCGGCAAAAATAA	155
	R: CCACACCCAATCCACCTGTT	

## Data Availability

The transcriptome data in this study are available from the NCBI SRA database under accession no. GSE249347.

## Supplementary Materials

The following supporting information can be downloaded at https://www.mdpi.com/article/10.3390/ijms25105096/s1.
