# Supplementary material for: DNA Methylation Analysis of Growth Differences between Upright and Inverted Cuttings of Populus yunnanensis
Source: Int J Mol Sci. 2024 May 7;25(10):5096. doi: 10.3390/ijms25105096 (PMC11121305; doi:10.3390/ijms25105096)
Supplement: Supplementary file 1 [file ijms-25-05096-s001.zip › ijms-2909285-supplementary.pdf]

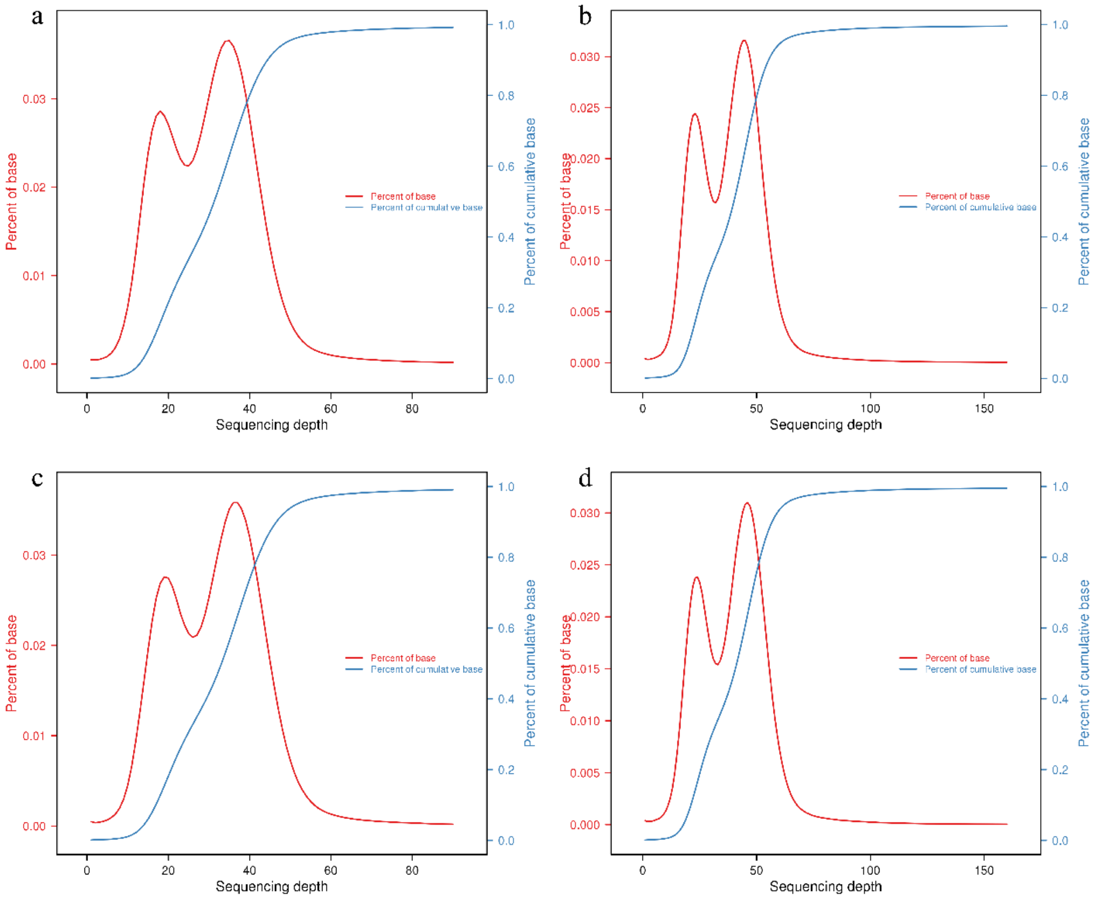

Figure S1. Sample sequencing depth distribution. a: JD; b: JZ; c: SD; d: SZ.

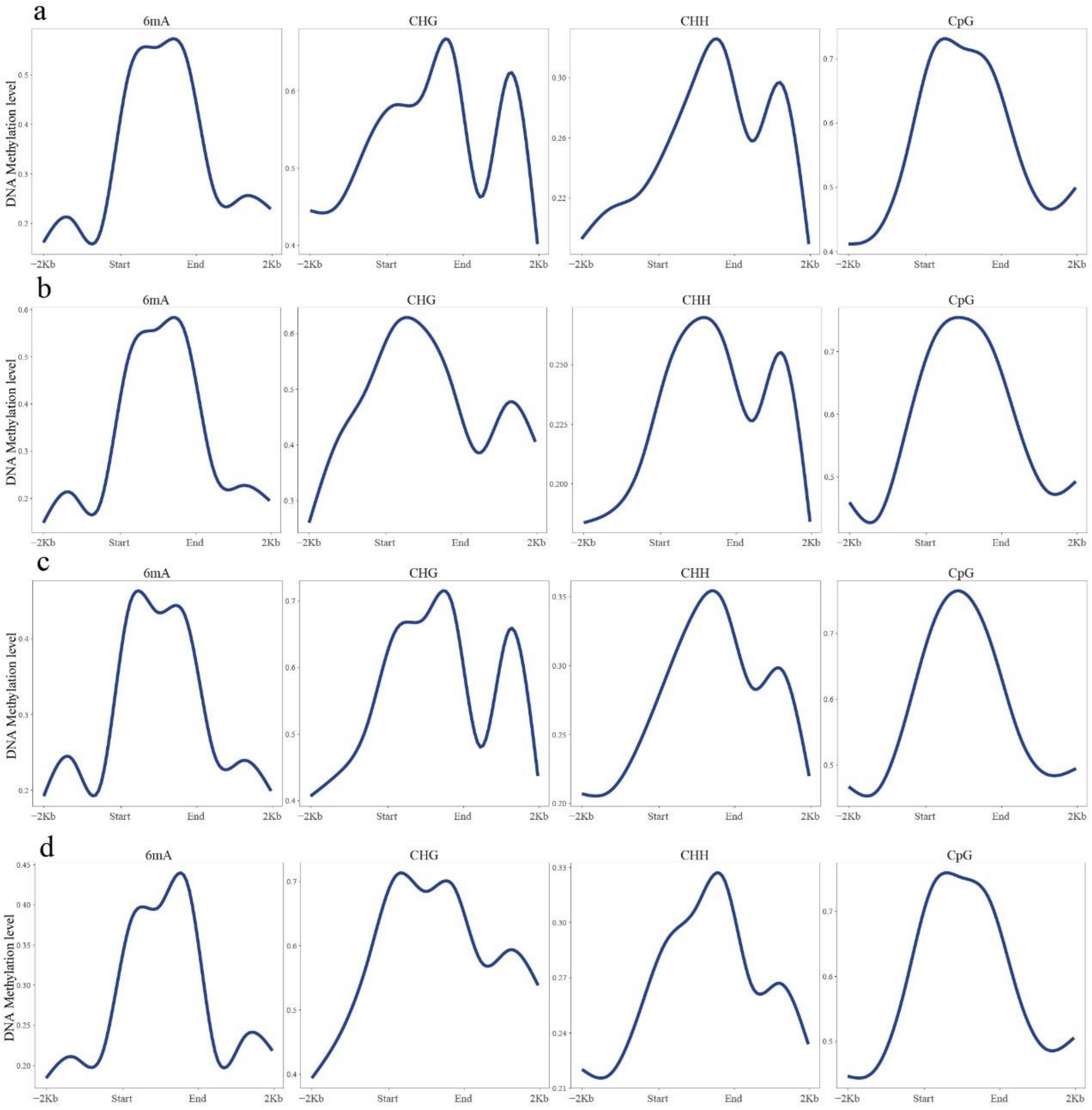

Figure S2. Repeat region methylation levels for each sample. a: JD; b: JZ; c: SD; d: SZ.

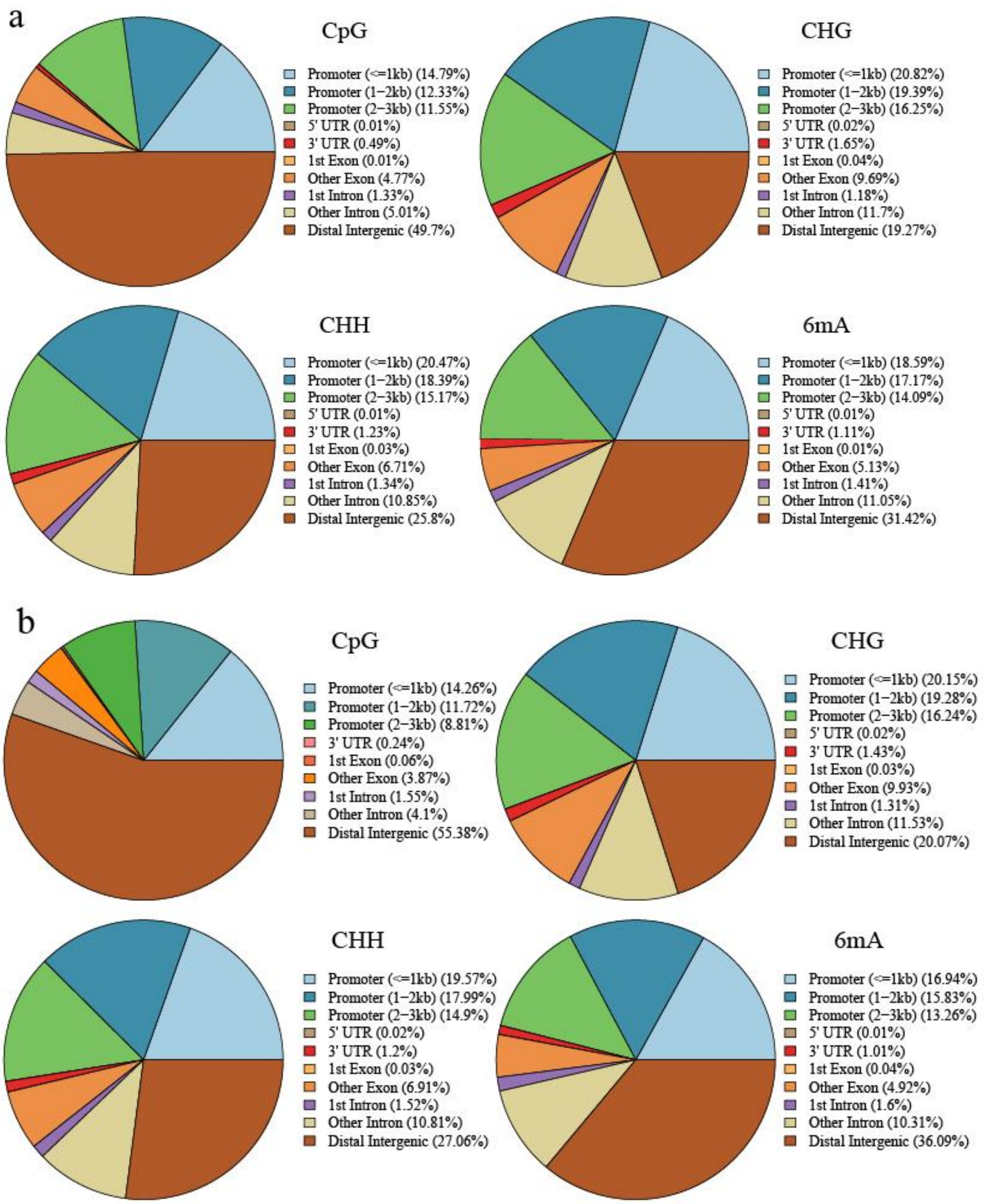

Figure S3 (A). DML gene region annotation classification. a: JZ_vs_JD; b: SZ_vs_SD.

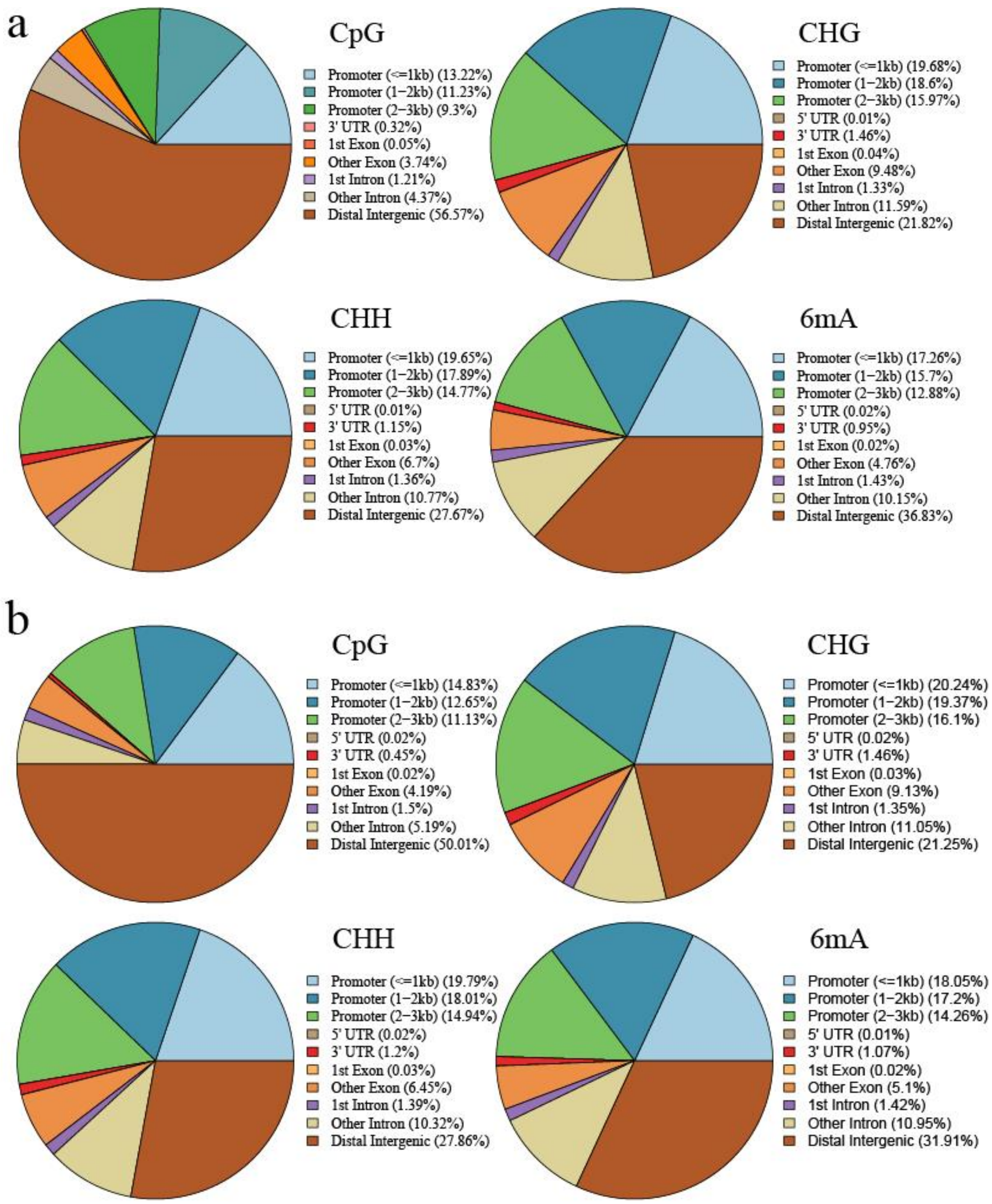

Figure S3 (B). DML gene region annotation classification. a: JD_vs_SD; b: JZ_vs_SZ.

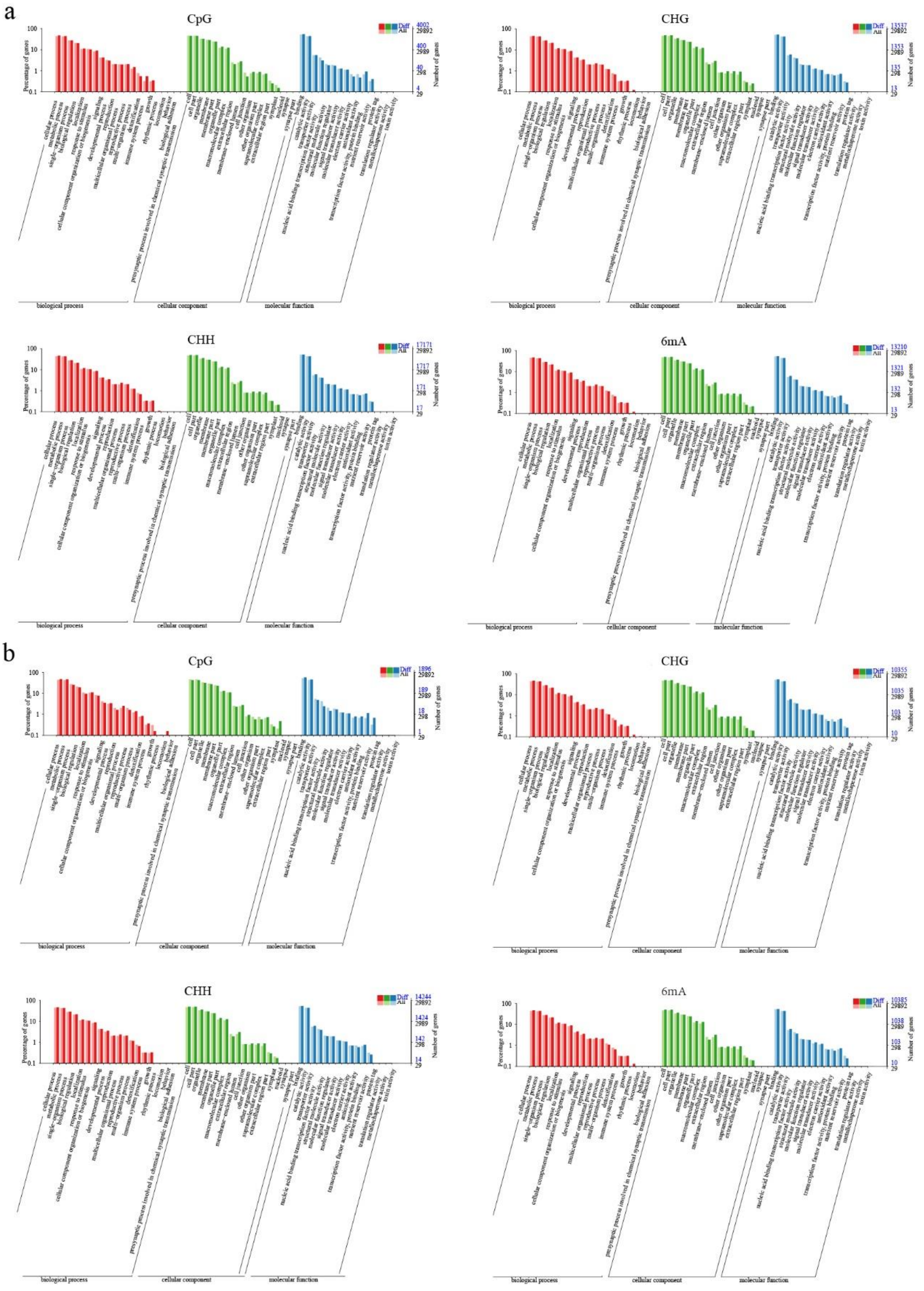

Figure S4. GO enrichment map of DMR-associated genes. a: JZ_vs_JD; b: SZ_vs_SD.

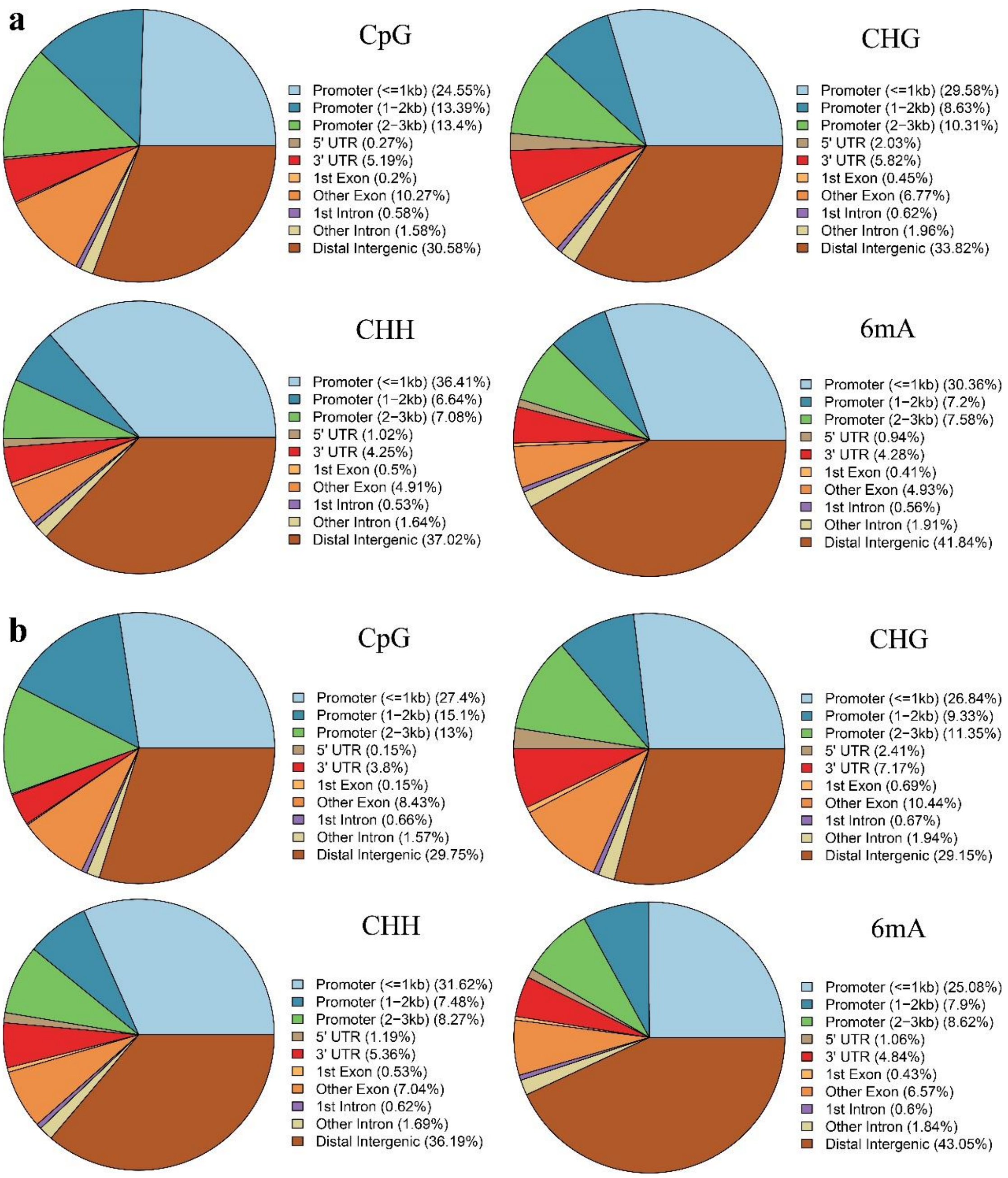

Figure S5 (A). DMR gene region annotation classification. a: JZ_vs_JD; b: SZ_vs_SD.

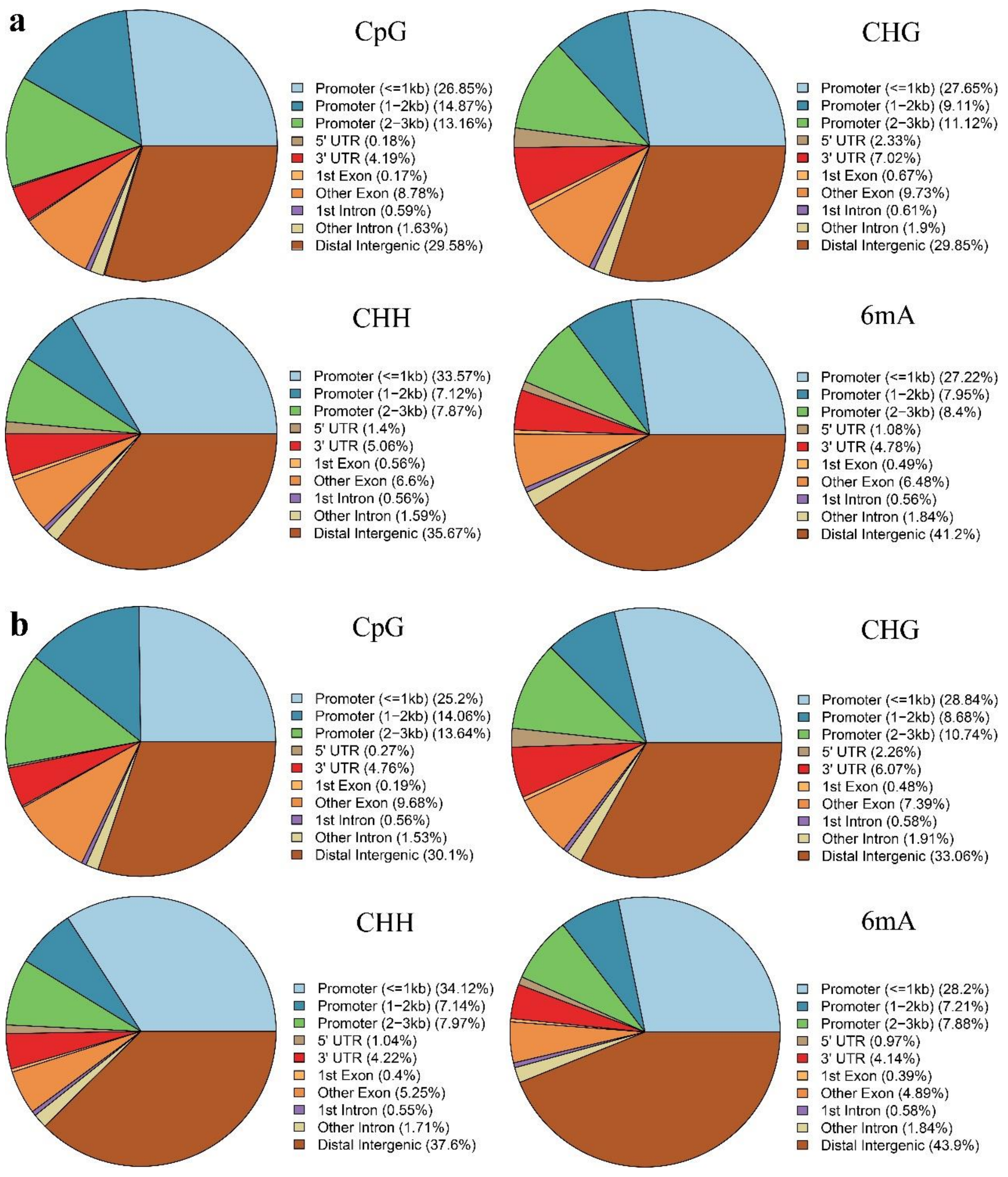

Figure S5 (B). DMR gene region annotation classification. a: JD_vs_SD; b: JZ_vs_SZ.
